# Case of Lithotomy in the Female, with Remarks

**Published:** 1845-07

**Authors:** A. Baker

**Affiliations:** Norwich, Chenango County, N. Y.


### RECORD OF MEDICAL SCIENCE.

*Case of Lithotomy in the Female, with remarks; reported by* A. BAKER, Jr., M. D., of Norwich, Chenango County, N. Y.—Mrs. N——, of New Lisbon, Otsego county, N. Y., aged forty-nine, had been laboring under symptoms of urinary derangement for the last thirty years. I first saw her in company with Dr. Henry Mitchell, on the 5th of February last. On our arrival she was in bed, with her face downwards, supporting herself on her hands and knees—when she gave us the following history. About thirty years ago, she was attacked with an urinary difficulty, characterised by frequent calls to micturate, pain in voiding the urine and a continual sense of irritation about the neck of the bladder. After rest, and taking diuretic medicines, she obtained partial relief, but from that time to the present she has hardly been free from irritation of those parts, at times very severe. About eleven months ago, her symptoms became very much aggravated, her calls to micturate much increased in frequency, and the pain, particularly at the close of the operation, very severe. She was now unable to relieve the bladder except in certain positions of the body, and that in the one already described, namely, on the hands and knees. For the last six months she has not left her bed without help, and even then, under the most excruciating pain, and her position in bed has been almost exclusively confined to her hands and knees. She now voids her urine in a few drops at a time as often as four or five times an hour, and labors under continual irritation and pain. The urine on examination, was largely mixed with blood and mucus, which she informed us had been almost constantly the case for nearly a year, though materially increased for the last six months. She still continues her menstrual evacuations with considerable regularity. Her bowels, though inclined to costiveness, generally yield to mild laxatives and regular diet, her pulse about eighty-five, irritated but not hard, her appetite and digestion tolerable, the respiratory organs in good condition.

Under this state of symptoms, we of course had no hesitation in pronouncing it a probable case of stone in the bladder. Fortunately in this we have a sure means of diagnosis, that does not pertain to many other departments of surgery. The sound was introduced and the presence of stone clearly indicated. It may not be improper here to give a description of the condition of the parts. The urethra, including the meatus urinarius and neck of the bladder, was in a highly irritable condition; so much so, that notwithstanding the utmost caution in introducing the sound, it gave her great pain. The neck of the bladder was so very sensitive that she could not be prevailed upon to change her position from her hands and knees during the operation of sounding. This position, with her shoulders lower than her hips, dropped the stone below the axis of the sound, so that in this position it could not be felt. We therefore elevated the shoulders with the greatest caution, which brought the stone in reach of the sound, hence the case presented itself in its true character.

Next for its removal. It is well known there are two *principal* operations for extracting the stone from the female bladder, viz: by dilatation of the urethra and neck of the bladder, or by incisions into the same structures. The former of these was judged to be impracticable from the extreme irritability of the parts. As her catamenial return was expected the following week, and as the parts were at present so exceedingly irritable, it was thought prudent at least to defer the operation. We accordingly put her under the free use of the carbonate of soda, and advised the topical use of anodyne fomentations, together with a daily introduction of the catheter, with the view of controlling the high sensibility of the parts.

On the 20th of February, we visited her again. She had been through the catamenial flow—had taken the soda—had used the fomentations, but the catheter she had not been able to use on account of the acute sensibility of the urethra. She was able to change her position a little more than on the former visit, for instance, she could lie on her back for a short time.

*Operation.*—She was placed on the table and secured in the common way, A straight grooved staff was introduced through the urethra and the stone felt with the beak of the instrument. The incision was now commenced by dividing the urethra half an inch posterior to the meatus urinarius with a sharp pointed bistoury, into the groove of the staff. The beak or probe-point of the lithotome knife was introduced through the opening thus made in the urethra into the groove of the staff and pushed forward along the groove, in a direction obliquely outward and downward, until it penetrated the bladder and the incision continued until of sufficient size to admit the forceps. The knife was now withdrawn and the forceps introduced. A stone about the size of a nutmeg was grasped and drawn out. The search was resumed and another stone secured and removed, and so on to the number of ten, varying very little in size. No more stones being found, the bladder was faithfully washed out with warm milk and water and the patient put to bed. She bore the operation well and expressed herself as being freer from pain than she had been in six months. She could now lie on her back or any other position that was proper. We found the walls of the neck of the bladder very much thickened. This was probably the result of the long continued irritation from the friction of the stones.

The after-treatment consisted in mild diet, gentle laxatives, diluents and fomentations and at the end of two days the introduction of the catheter, which was suffered to remain for two weeks, with the exception of its being occasionally removed for the purpose of cleansing. At the end of this time the wound was found to be nearly healed and the catheter was permanently removed. After the removal of the instrument she was able to retain her urine for two hours or more, and exercise full control over its evacuation. Nothing of an unpleasant character occurred till a day or two previous to the removal of the catheter, she swallowed by mistake about two drams of tartaric acid supposing it to be Rochelle salts. This produced severe gastric disturbance, consisting of flatulence, nausea, vomiting, &c. The severity of these symptoms, however, was soon allayed and she continued to progress in the most favorable manner so far as the urinary difficulty was concerned. Her digestion, however, continued weak, her nervous system became irritable, her nights restless, her mind despondent and foreboding and at the end of about three months, she detected a white sediment in her urine. This increased her alarms and apprehension of a return of her former difficulty. She finally voided, with considerable pain, a crystallized white mass of some fifteen grains weight and irregular form. This she sent to Dr. Mitchell and myself for our examination and advice. We found on analysis it consisted of the triple phosphate of magnesia and ammonia, whilst the former *calculi* consisted mainly of lithic acid. We attributed her present sufferings together with the deposition in her urine to weakness of digestion and general nervous irritability. We therefore advised opiates, tonics and exercise and a more nutritious diet as soon as her stomach would bear it. Under this treatment she soon improved, and the deposition in her urine ceased.

During the presence of a deposite in her urine, she was annoyed with more frequent calls to micturate, yet even then she had full control over the evacuation to retain it and pass it at will From this time her general health steadily improved and she is at this time, as I understand, in the enjoyment of very good health and able to retain her urine the ordinary length of time, and is not at all troubled with incontinence.

The satisfactory result of this case is to be attributed fully, as I think, to the mode of performing the operation, namely, to leaving the meatus and anterior portion of the urethra undivided. Where dilatation of the urethra is practicable, it should undoubtedly be chosen as the simpler method, but even in *this*, incontinence of urine is a frequent consequence. Unfortunately, however, in the majority of cases, especially those of long standing, there is so much irritability of the parts as entirely to preclude the success of this operation. Such was the fact with the case under consideration. It is well known to the profession, that the common operation of lithotomy in the female, namely, of dividing the urethra its entire length is almost invariably followed by incontinence. Such at least appears to have been the experience of Sir Astley Cooper, both in his own operations, and those he witnessed in others. He holds the following language in relation to this operation: "In all the cases of this operation that I have performed or witnessed, the urine has not afterward been retained, but I would not deny that a patient might recover the retentive power. As the loss of retention is a greater evil than I can describe, producing excoriation and a very offensive state, I shall in any future operation of lithotomy, try what may be effected by employing a suture to bring the divided parts together."

Such seems to have been the experience of one of the greatest operators the world ever produced, and can men of less pretensions expect better results in the old way of operating. The advantage of leaving the commencement of the urethra uncut, will be apparent to every one, and of course needs no particular explanation. It at least acts the part of Sir Astley's proposed suture, without the risk of its irritating or being torn out, not to speak of the pain and difficulty of its application. The only precaution I consider necessary to secure its success, in addition to the common position and care, is the continued use of the catheter, to prevent the urine flowing through the wound. The two extremities of the wound being secured by natural structure, and the wound being kept free from urine by the use of the catheter, there is nothing to prevent its rapidly and permanently healing as in other parts. This operation so far as I know was originated by Doctor Mitchell.—*Trans. N. Y. State Medical Society.*

